# Numerical Analysis of Experimental Research in a Lightweight Floor System (LFS) with Heat Diffuser

**DOI:** 10.3390/ma15186466

**Published:** 2022-09-17

**Authors:** Karpiesiuk Jacek, Tadeusz Chyży

**Affiliations:** 1 Elektra Kardo, Produkcyjna 59/1, 15-680 Bialystok, Poland; 2 Faculty of Civil Engineering and Environmental Sciences, Bialystok University of Technology, Wiejska 45A, 15-351 Bialystok, Poland

**Keywords:** lightweight floor system, aluminium lamellae, polyurethane adhesive, strain gauge techniques, floor’s stresses, thermal action

## Abstract

The article presents the results of research on a lightweight floor system (LFS) with a heat diffuser made of metal lamellae. It differs from traditional layered floors in the absence of a screed layer, which reduces thermal inertia and predisposes it to be used with renewable energy sources. As part of the research, a real model of the floor, consisting of nine ceramic tiles, was made. Polyurethane adhesive was used to connect the individual layers of this composite. The model was subjected to a thermal action. It was constructed with the measuring equipment consisting of strain gauges. These were located at the boundaries of the composite layers and measured the material’s deformation. The measurement results were verified by numerical calculations. For this purpose, a computational model was made using FEM (finite element method). Comparable results of deformations were obtained (the differences did not exceed 6.1%), which made it possible to perform numerical calculations of light floor materials stresses. Additionally, the displacement of the tested model was measured and numerically verified. The results of these verifications can be useful not only in the heated/cooled LFS with aluminium lamellae, but also in other building partitions inside and outside the building.

## 1. Introduction

In the modern world, there is an urgent need to search for technical material solutions conducive to the use of energy from renewable sources, as well as technologies that reduce its consumption. This topic is taken up by scientists and such problems can be found, for example, in [1,2,3,4]. Hence the need to use more efficient heaters. One solution could be floor or wall-mounted radiators. Heaters of this type have a layered structure with a built-in heating element—most often in the form of a heating coil or electro-resistance structures. Renewable energy sources are dependent on the weather. Therefore, it is justified to use heaters that use even small energy resources. Lightweight floor systems (LFS) are such a solution. These systems eliminate the energy accumulator, which is the heavy concrete slabs (screed). The floor layer (ceramic tiles) with an insulating layer was combined with the use of polyurethane adhesives, which spread easily and make the structure more flexible when exposed to changing temperatures. The use of this type of adhesive is also justified due to the good properties of mechanical compatibility. It is characterized as the product of the thermal expansion coefficient α multiplied by Young’s modulus of elasticity *E*. Polyurethanes have a low *TN* value due to low *E*-values, despite high α values. Therefore, the thermal stresses arising in them do not cause the destruction of less-resistant substrates, and besides, they are able to withstand much greater deformations than materials with significant stiffness. The proper connection of layers with different mechanical parameters was dealt with in [5,6,7]. The topic of stress reduction using the dependence of dynamic and static modules, when there are temperature and pressure changes, and when obtaining an elastoplastic solution of stresses and strains while also dealing with the influence of heterogeneity in mechanical parameters, can be found in articles [8,9]. The above-mentioned works contributed to the introduction of polyurethane adhesives into the research. Thermal efficiency is to be increased by the used aluminium diffuser, the so-called lamellae. Aluminium was chosen because of its high thermal conductivity parameters and good cooperation in the well-matched polyurethane adhesives. The presented technical solution was subjected to experimental tests to determine the value of internal forces—stresses, in conditions similar to operational ones. As part of the research, a floor fragment, with dimensions of 3 × 3 ceramic tiles, was made. The model was subjected to a thermal action corresponding to the assumed average operating temperatures. The floor model was made together with the measuring equipment composed of strain gauges. The strain gauges were placed inside the composite at the boundaries of individual layers. Additionally, the digital image correlation (DIC) system was used to measure the model displacements. The results were checked by making a calculation model using FEM. Comparable results of deformations were obtained, which made it possible to perform numerical calculations of stresses inside the lightweight floor. The material parameters adopted in the computational analysis are summarized in Table 1. They are taken from the research included in [10,11,12,13,14,15,16,17,18,19,20,21,22].

Strength data and material parameters of the polyurethane adhesives used, such as Young’s modulus *E*, Poisson number *ν*, and thermal expansion coefficient *α*, were obtained from the tests performed by Karpiesiuk [23,24]. The obtained results will be summarized and compared, which will allow for an assessment of the test’s correctness.

## 2. Materials and Experimental Methods

**Figure 1:**
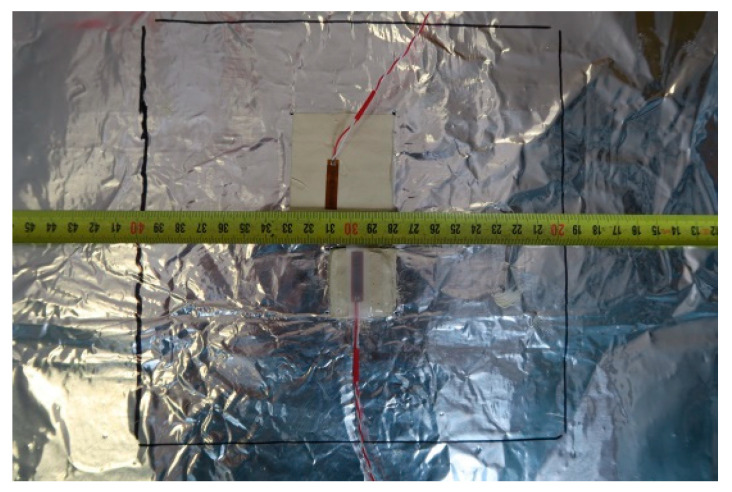
Attaching the strain gauges to polyurethane adhesive, with lamellae in the centre of the model, the field dimension 20 × 20 cm.

In order to carry out a numerical analysis of the LFS tests with aluminium lamellae, immediately after determining the material parameters of individual layers of this lightweight floor type, it was decided to experimentally check the size of deformations and displacements caused by temperature. For this purpose, a model of a light heated floor with a heating coil and a heat-dissipating layer—lamellae—was prepared. The research was conducted at the Bialystok University of Technology with the use of strain gauge and digital image correlation (DIC) techniques. The set of DIG system devices is used for non-contact, three-dimensional measurements of deformations caused by static and dynamic loads. It is based on a series of photos taken with digital cameras to recognize the surface structure of the measured object (each pixel in the photo is assigned appropriate coordinates). The purpose of this method is to analyse, calculate displacements and document the deformation of the measuring material. Graphical presentation of the research results obtained enables a complete understanding of the behaviour of the tested object. The measuring stand was 60 × 60 cm, and the floor was covered with 20 × 20 cm tiles. Active strain gauges were placed at different levels of the system layers, between the central tile plate and the XPS. Before embedding, the strain gauges were attached to previously prepared substrates of the materials used in the test. Figure 1 shows the place of the strain gauges installation on the polyurethane adhesive.

**Figure 2:**
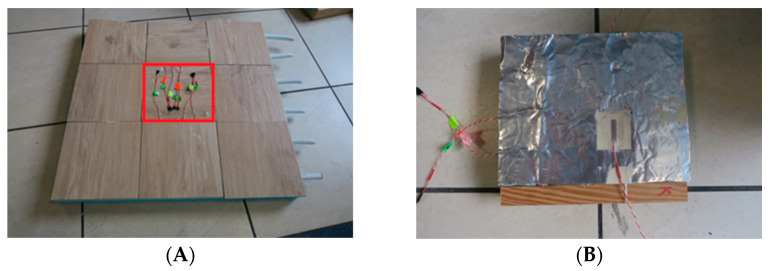
Research model with polyurethane adhesive and lamellae: (**A**) experimental model 60 × 60 cm with measurement site 20 × 20 cm (in red); (**B**) small composite 20 × 20 cm.

The experimental model of the lightweight floor consisted of 40 mm XPS thermal insulation with grooves where a 16 mm diameter heating coil was placed. The heating pipes were not fed with water, but with an Elektra UltraTec heating cable with unit power of 10 W/m. An aluminium lamella with a thickness of 0.06 mm with a 1 mm layer of Sika Force-7710 L100 polyurethane adhesive was used. The aluminium lamella was covered with 8.5 mm thick Tero ceramic tiles by Paradyz, fixed with Sika BondT8 polyurethane adhesive. The joints between the ceramic tiles were filled with C2S1 flexible cement mortar. The experimental model, with a similarly constructed smaller composite with dimensions of 20 × 20 cm, (necessary for the placement of passive strain gauges) is shown in Figure 2.

Registration of the deformations and the temperature in the centre of the composite were determined according to the description in the article [25]. Additionally, in the same way, as described in [25], the deformations of seven active and passive strain gauges, installed on each of the LFS layers, were recorded.

**Figure 3:**
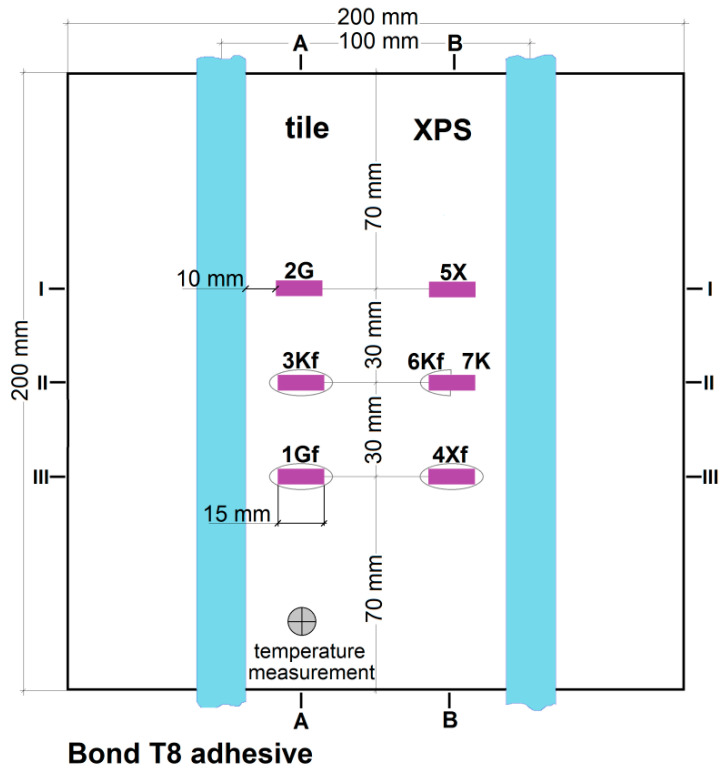
Horizontal section of the test model in the centre with polyurethane adhesives, with lamellae.

**Figure 4:**
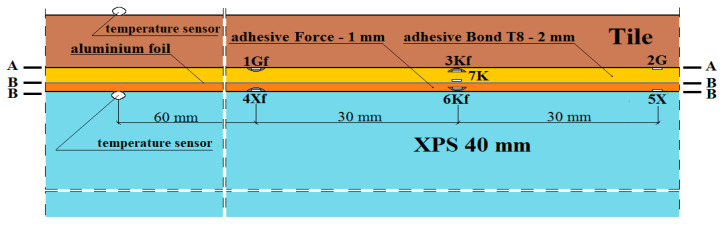
A and B vertical sections of the research model with polyurethane adhesives, with lamellae.

**Figure 5:**
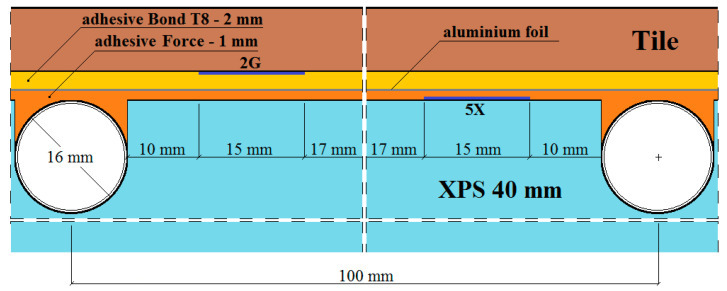
Vertical section I–I of the research model with polyurethane adhesives, with lamellae.

**Figure 6:**
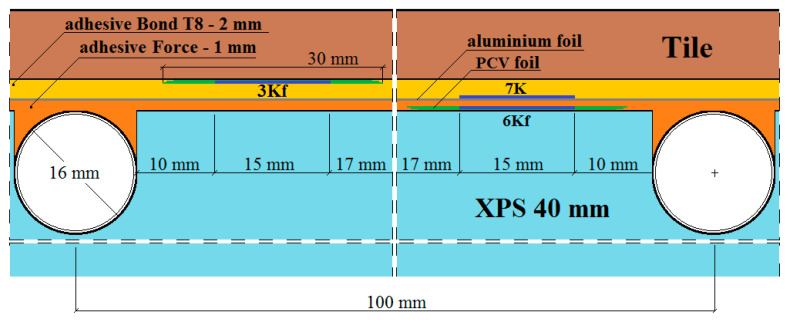
Vertical section II–II of the research model with polyurethane adhesives, with lamellae.

**Figure 7:**
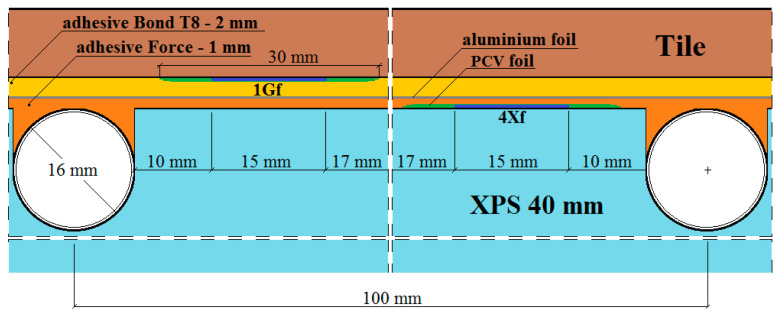
Vertical section III–III of the research model with polyurethane adhesives, with lamellae.

The places of installation of the active strain gauges and temperature sensors in the central part of the model are shown in Figure 3, Figure 4, Figure 5, Figure 6 and Figure 7.

In Figure 3, Figure 4, Figure 5, Figure 6 and Figure 7:
1*G_f_*—strain gauge on the tile, separated from the adhesive by a PVC foil,
2*G*—strain gauge on the tile, covered with adhesive,
3*K_f_*—strain gauge on the adhesive, separated from the tile by a PVC foil,
4*X_f_*—strain gauge on the XPS, separated from the adhesive by a PVC foil,
5*X*—strain gauge on the XPS, coated with adhesive,
6*K_f_*—strain gauge on the adhesive (from the bottom of the aluminium foil), separated from the XPS by foil,
7*K*—strain gauge on the adhesive, covered with adhesive, placed transversely to the heating coil.

**Figure 8:**
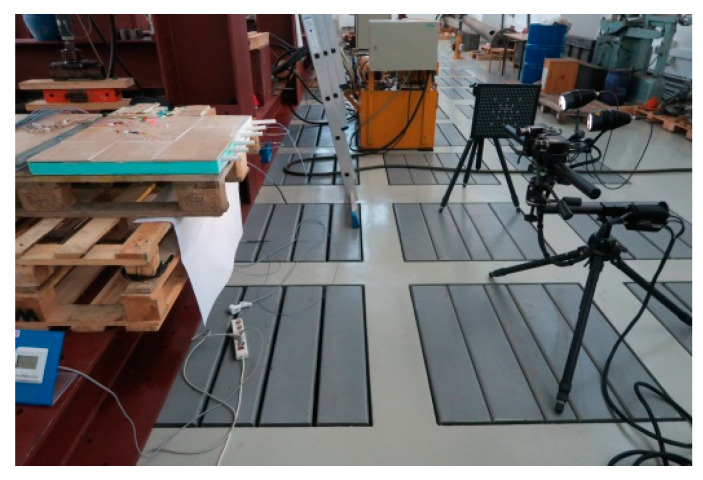
Digital image correlation system (DIC) for measuring LFS displacements.

Besides the deformation research, the displacement of the test model under the influence of temperature changes and its own weight were also tested. For this purpose, the DIC—visual digital image correlation system implemented in the Aramis program was used. Figure 8 shows this collaboration and the stand for displacement test. The maximum width of the view field for the model displacement research was approximately 240 mm. The image was placed between the edge of the model and two points at the centre of the test model.

**Figure 9:**
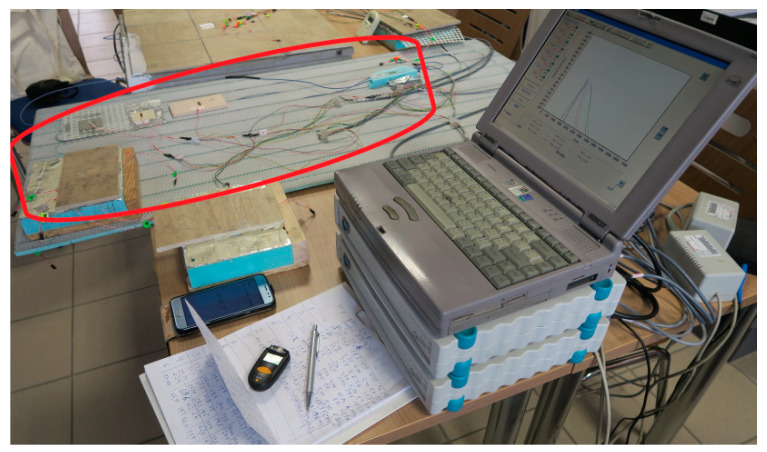
The stand and the research equipment for the correction of the system component materials.

A very important stage of the layers deformation research in the LFS system, using the strain gauge techniques, was the performance of correction tests. The correction of the initial deformation results is necessary when there are thermal interactions, which was confirmed in the studies [26,27]. For this purpose, the same measurement technique was used as in the investigated initial deformations, which is shown in Figure 9. During the correction of deformations, active strain gauges were placed separately on each LFS layer (not inside of the composite) and subjected to temperature action, and the passive ones were connected to the prepared previously smaller model with dimensions of 20 × 20 cm. During the research correction, the time of the temperature readings on the samples was synchronized with the results of deformations given by the test equipment.

## 3. Results and Discussion of Experimental Methods

**Figure 10:**
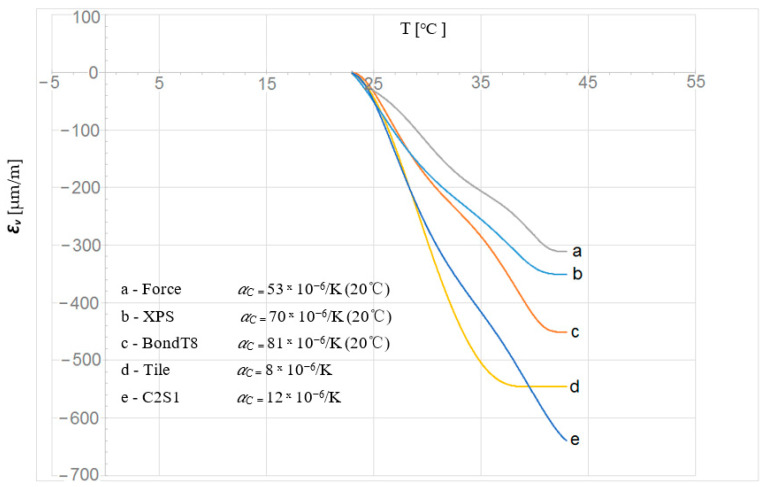
Temperature reactions of strain gauge deformation *Ɛ****_v_*** mounted on different measuring materials.

The process of research and calculations using the strain gauge technique was precisely described by Karpiesiuk and Chyzy in [25]. They proved that this type of research consists of several processes. Their reason is the temperature variation during the tests. Therefore, reading only the initial results, without their correction, should be treated as an incomplete research cycle. As proved in [25], this correction, especially when dealing with elastic materials, e.g., polyurethanes, gives the best results when the reaction of strain gauges to temperature (apparent strain) is achieved through experimental studies. In this way, we get different apparent strain curves *ε_v_*. Examples of temperature reactions are shown in Figure 10. These reactions were achieved through additional experimental studies, starting at 23 °C. They were used to correct the initial deformation results by subtracting from the initial results, obtained from the correction.

The theoretical Equation (1) can also be used to correct the deformation. Based on the theoretical Equation (1) and experimental correction tests (Table 2 and Table 3), the deformation correction results were prepared, respectively. The analysis of the deformation results obtained computationally and experimentally confirmed that they can be similar (values and signs −/+) only for “rigid” materials—ceramic tiles. This means that the appropriate decision for LFS, made of elastic polyurethane adhesives, is acceptation only the results of the experimentally obtained correction.

*Ɛ_v_* = (*α_R_*/*k* + *α_C_* − *α_M_*) D*T* (1)

where *Ɛ_v_*—the temperature reaction of the strain gauge (the temperature correction), *k* = 2.19—the strain gauge constant, *α_R_* = 0.49 (Ω × mm^2^/m) or (−60)—(−80) × 10^−6^ [1/K], *α_M_* at 20–100 °C = 13.5 × 10^−6^, CuNi44—Konstantan at 20–200 °C + Isotan foil (data from the manufacturer –Tenmex), and *α_C_*—the thermal expansion of the constituent material.

According to Equation (1), the corrections of the strain gauges used at the reference temperature, e.g., 20 °C, depending on the adopted materials, will take the following values:

*Ɛ_v_._tile_* = (−60/2.19 +8 − 13.5) × 10^−6^ × D*T* = −33 × 10^−6^ D*T*,
*Ɛ_v_._BondT8_* = (−60/2.19 +81 − 13.5) × 10^−6^ × D*T* = +40 × 10^−6^ D*T*,
*Ɛ_v_._Force_* = (−60/2.19 +53 − 13.5) × 10^−6^ × D*T* = +12 × 10^−6^ D*T*,
*Ɛ_v_._XPS_* = (−60/2.19 +70 − 13.5) × 10^−6^ × D*T* = +29 × 10^−6^ D*T.*

Initial deformation results for LFS constituent materials with lamellae are placed in Table 4. These results were then corrected based on experimental tests. The final results are listed in Table 5. The deformations given are for the central zone (the ceramic tile centrally located).

In one place, the 5X strain gauge on the XPS, coated with adhesive, no deformation correction was made, considering the experimental data as unreliable. The reason for the measurement error could be incorrect readings of strain gauges in the places of two-sided to polyurethane adhesion (which were not separated by a PVC foil).

(2) $$T_{w}=\frac{U_{1\;}\cdot \;T_{kl}+U_{2}\;\cdot \;T_{po}}{U_{1}+U_{2}}$$

where

*U*_1_—heat transfer coefficient in the adhesives;
*U*_2_—heat transfer coefficient in the ceramic tiles;
*T_kl_*—adhesive temperature above the thermal insulation (reading from the sensor);
*T_po_*—temperature on the ceramic tile.

**Figure 11:**
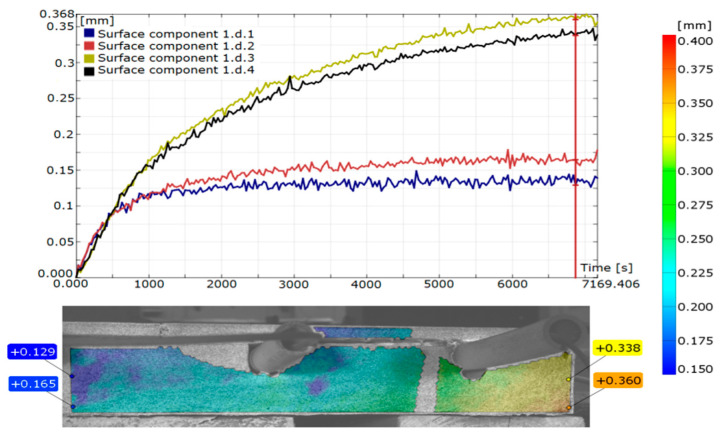
The displacements between the centre and the edge in LFS with lamellae.

The values and manner of displacement were measured using the digital image correlation (DIC) method on a span of 232 mm and shown in Figure 11. Displacement measurements were made 7000 s after the start of the test, and their average result was 0.209 mm (0.338–0.129 mm). These values were read when the displacements stabilized. The data in Table 5 show that all materials expand under the influence of temperature. Verification of the deformation value and displacement (deflection) should be confirmed by building the FEM calculation model, using the same conditions and places of installation of strain gauges.

## 4. Results and Discussion of Experimental Methods

**Figure 12:**
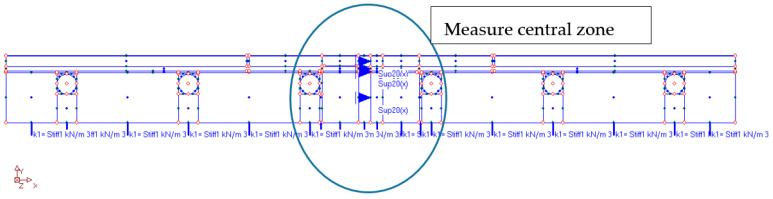
The computational model of the studied structure.

**Figure 13:**
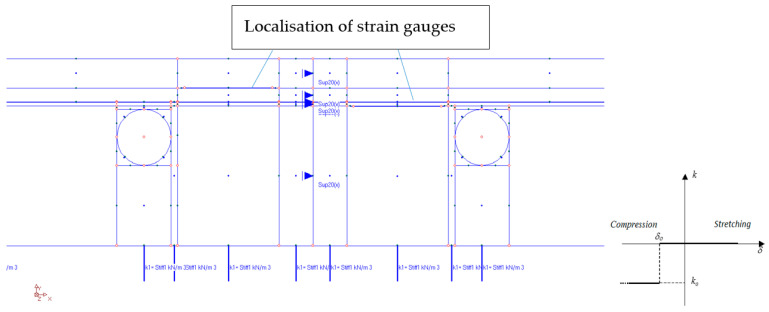
Measurement zone and substrate modelled by “contact zone”. *k, k_o_*—spring stiffness; *δ, δ*_o_—displacement.

For this part of the research, the “ORCAN” structure analysis system, version 0.98 was used, which was fully implemented at the Bialystok University of Technology. Structural modelling in this system was carried out through the implementation of procedures and algorithms of the Finite Element Method (FEM). The computer implementation was made with the use of C and PASCAL compilers. Figure 12 shows the mapping of the experimental model where the displacement and the deformation on the edges of the central zone were measured. The materials used in this model were precisely described (together with thickness) in the second part of this paper. This model consisted of these materials, starting from the top side: ceramic tiles, Sika BondT8 polyurethane adhesive, aluminium lamella, Sika Force-7710 L100 polyurethane adhesive, and XPS thermal insulation lying freely on the concrete. The distribution of the measuring strain gauges is shown in Figure 13. The joints of C2S1 have been designed between the tiles. For verification of the experiment, the temperature data at 7000 s from Table 4 and Table 5 were used: on the external surface of the tile −8.5 °C; on the joint between the ceramic tile and adhesive BondT8 −12.0 °C; and at the interface of adhesive Force and XPS insulation −16.6 °C. The installation temperature was according to Table 4 and Table 5, namely *T_o_* = *T_i_* = 23.4 °C. The model was loaded with self-weight only. The research model was placed freely on the substrate. This substrate was modelled with a so-called “contact zone”, which works like a spring. In the computational analysis of this variant, the friction force was omitted. It was assumed that the floor was laid freely on a concrete base.

**Figure 14:**
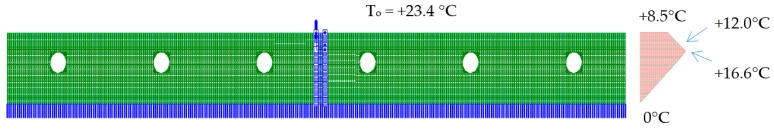
Model discretization with temperature action in the floor, length 60 cm.

**Figure 15:**
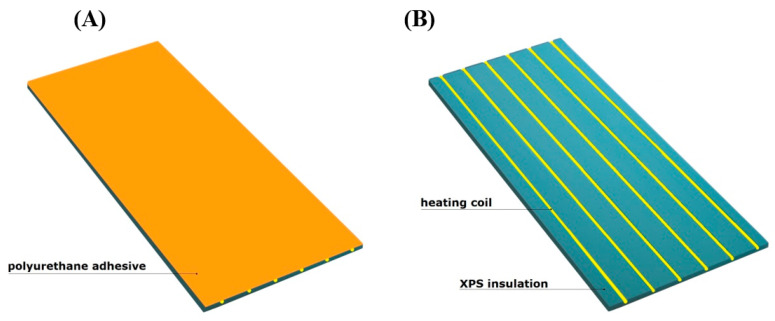
The 3D visualization: (**A**) the sample with a polyurethane adhesive; (**B**) heating pipes with 10 cm spacing.

The numerical model was built considering the cross-section through the centre of the test system. The plane stress state (PSS) working in a unit thickness of 1.0 m was used. This means that in the direction perpendicular to the separated band, the stresses are close to zero. Zero stresses were assumed in the direction perpendicular to the separated strip, which is a consequence of the lack of friction and other resistances caused, for example, by volumetric resistances related to the limited geometry of the room, or other resistances. The finite elements were rectangular shapes, dimensions 1 × 2 mm. Contact with the substrate was modelled with special one-dimensional spring-type finite elements, in which the stiffness change was used, depending on the direction of internal forces in the spring. The heating pipes’ dimensions of 0.3 × 0.3 mm were modelled, as the beam elements with the cross-section of the aluminium coil layer. Horizontal support was applied in the axis of symmetry (the blue arrows in Figure 12 and Figure 13) to stabilize the computational model. The division into finite elements with the temperature distribution, shown in Figure 14. Figure 15 shows the 3D model visualization.

**Figure 16:**
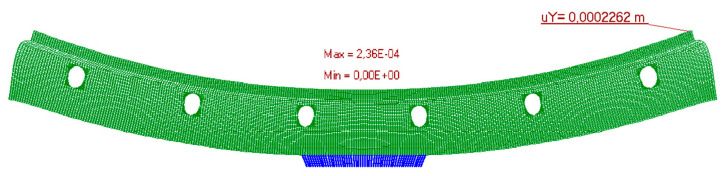
Deformation of the model.

**Figure 17:**
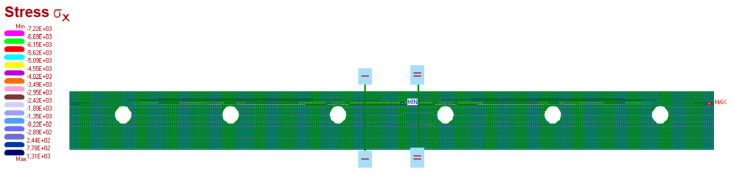
The map of horizontal stresses σ*_x_* and cross-section where the strain gauges are localised.

**Figure 18:**
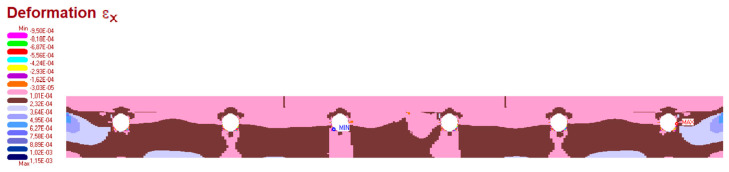
The map of horizontal deformations ε*_x_*.

**Figure 19:**
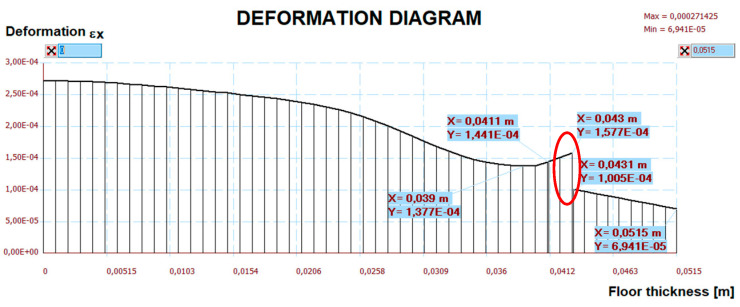
The diagram of horizontal deformations ε*_x_* in the I–I section.

**Figure 20:**
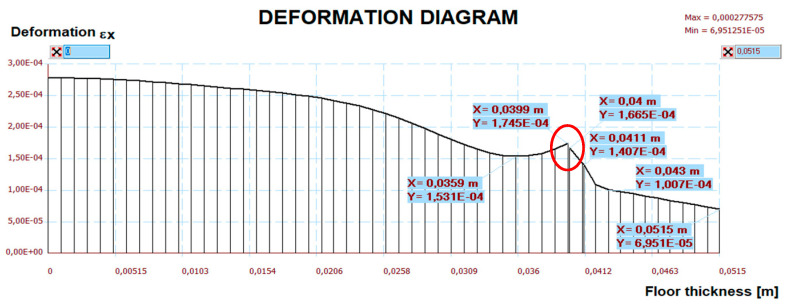
The diagram of horizontal deformations ε*_x_* in the II–II section.

The measurement results are set below (Figure 16, Figure 17, Figure 18, Figure 19 and Figure 20). Figure 16 shows the method of LFS deflection and displacement from thermal actions obtained through numerical calculations. The displacement and deformations from 7000 s of the experiment should be compared. The displacement at the edge of the model was 2.26 × 10^−4^ m. The map of horizontal stresses *σ_x_* is shown in Figure 17. The cross-sections I–I and II–II marked in this drawing pass through the strain gauges located in these places. The map of horizontal deformations is shown in Figure 18, Figure 19 and Figure 20 the results of deformation in cross-sections I–I and II–II are defined. The deformation of the materials to which the strain gauges are attached is marked in red.

Table 6 summarizes the results of deformations from experimental tests, comparing them with the results of numerical calculations and comments on them.

Relative differences, placed in Table 6, obtained from tests and calculations are not greater than 6.1%—when separating the strain gauges from the material with foils. Larger deviations occur in the place where the strain gauges are not separated from the layer material (point 2G). However, considering Zajac’s research thesis [28], this value can be assumed as reliable. The “too large” error occurs only at point 4*X_f_*, where the probable cause could be the imprecise sticking of the strain gauge. The displacements between the experimental and the computational version also differ slightly, only by 0.02 mm. The average result—for the tests is 0.205 mm, calculated is 0.226 mm. It can be concluded that small differences in the deformation and displacement of LFS with aluminium lamellae and polyurethane adhesives prove the correctness of the material parameters adopted for calculations, as well as the correct preparation of the research models.

**Figure 21:**
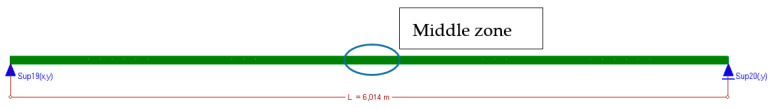
Numerical model of the reinforced concrete ceiling.

**Figure 22:**
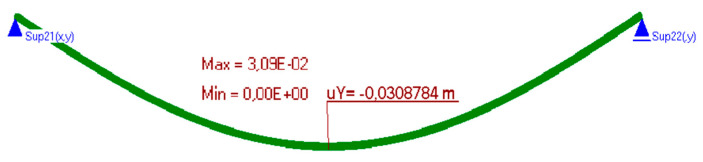
Deformation of the model.

Convergent results of the verification of experimental tests and FEM calculations allow to check the stresses and then relate them to the strength of materials used in LFS. For this purpose, numerical calculations with the finite element method were used. A reinforced concrete floor with a span of 6 m was adopted (Figure 21) with self-weight and imposed load, as well as non-static thermal actions, assumed from the experimental model. An extreme deflection was also assumed of the reinforced concrete ceiling with the value of 3.10 cm, in accordance with the standard [29] obtained after applying a uniformly distributed load of 30 kN/m^2^. The purpose of the numerical calculations was to check whether the stresses of the materials in the lightweight floor, where polyurethane adhesives were used, at various load combinations, including the maximum (according to the standards) ceiling deflection, would not exceed the strength. The assumptions for the calculations were as follows:

- The maximum deflection 1/200 from the standard [29] (assumed to be 31 mm; Figure 22).
- The lightweight floor model with polyurethane adhesive was repeatedly duplicated up to a ceiling span of 6.00 m.
- The reinforced concrete ceiling was connected to the thermal insulation with C2S1 adhesive.
- The reinforced concrete ceiling had a 20 cm of thickness.
- The joints were the same as in the experimental model, made from C2S1 adhesive
- The gaps, with thickness of 1 mm, between the XPS insulation were made.
- The aluminium foil was modelled separately in PSS.
- The self-weight, the imposed load of 2 kN/m^2^, and the thermal action were as in the experimental model.

**Figure 23:**
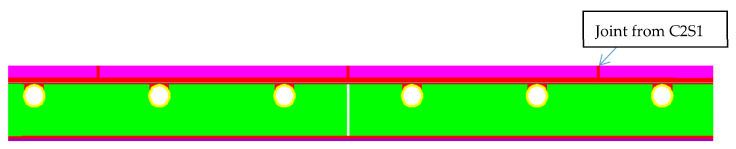
All floor layers in the middle zone.

Figure 23 shows the middle zone with all floor layers and the joints.

**Figure 24:**
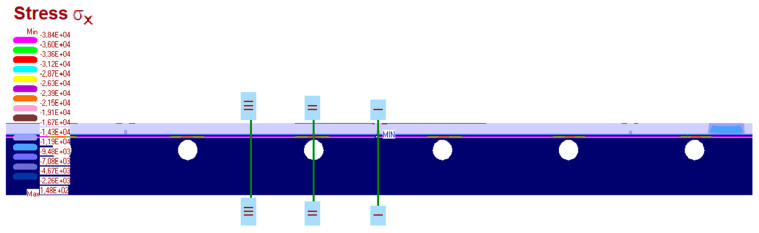
The map of the horizontal stresses σ*_x_* in the middle zone with three cross-sections.

**Figure 25:**
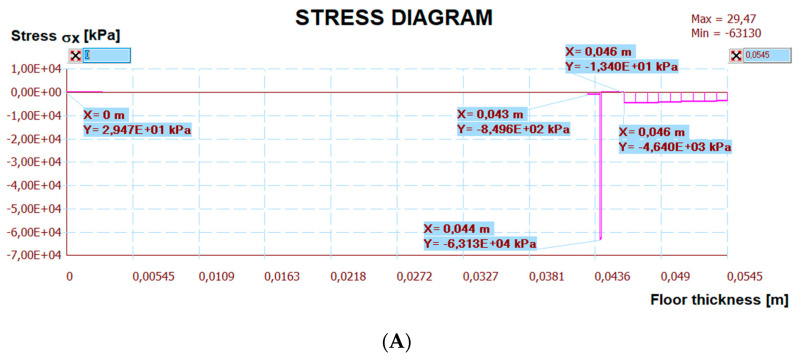

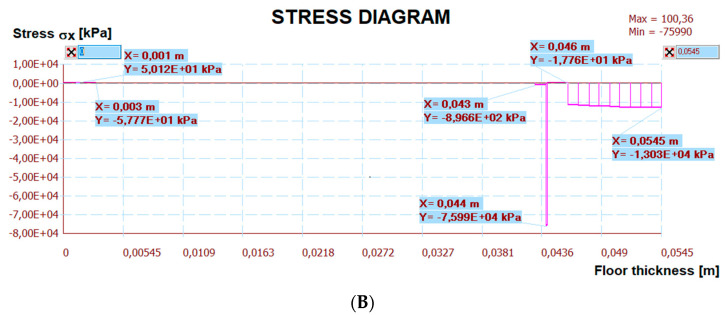
The diagram of horizontal stresses σ*_x_* in the I–I section: (**A**) thermal action without ceiling deflection; (**B**) thermal action and imposed load with ceiling deflection.

**Figure 26:**
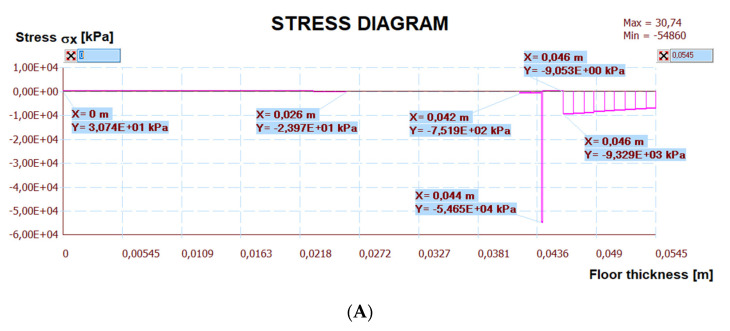

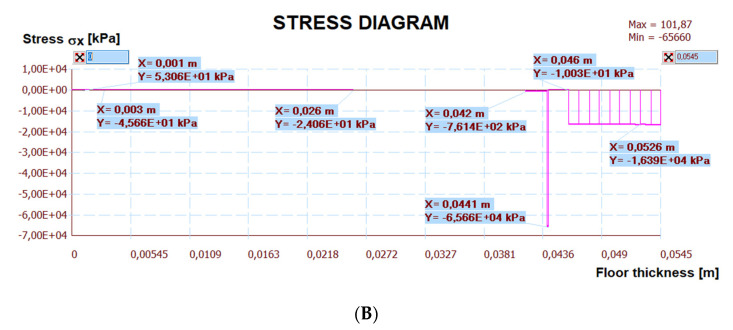
The diagram of horizontal stresses σ*_x_* in the II–II section: (**A**) thermal action without ceiling deflection; (**B**) thermal action and imposed load with ceiling deflection.

**Figure 27:**
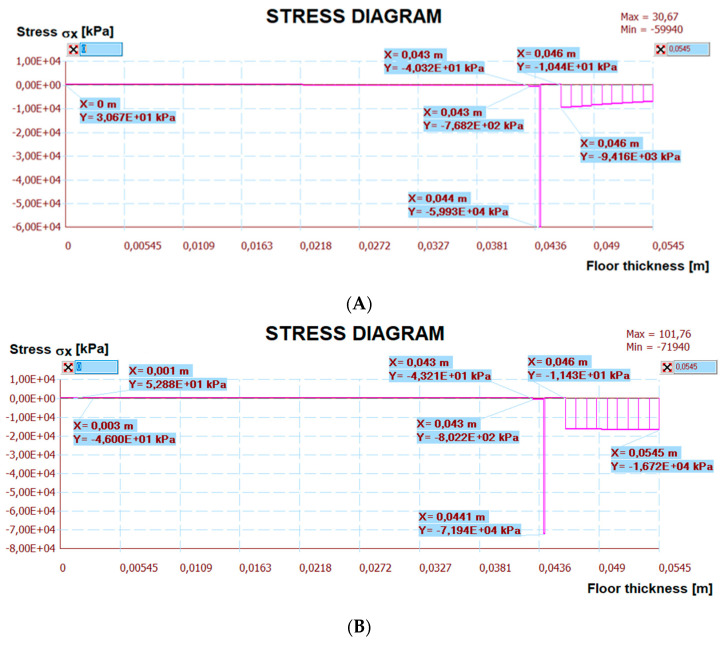
The diagram of horizontal stresses σ*_x_* in the III–III section: (**A**) thermal action without ceiling deflection; (**B**) thermal action and imposed load with ceiling deflection.

Figure 24 shows a map of the horizontal stresses with three sections. The calculation results are presented in Figure 25, Figure 26 and Figure 27. The I–I cross-section was located at the gap between the thermal insulation plate connections, which also passed through the tile joint. The II–II cross-section ran through the heating pipe, and the III–III cross-section transected all floor layers with lamellae. Figure 25, Figure 26 and Figure 27 show the results of horizontal stresses in these three sections, depending on the following loads:

(a) Thermal interaction plus the floor self-weight, without the maximum standard deflection of the reinforced concrete ceiling.
(b) Thermal action plus the floor self-weight and imposed load with the maximum standard deflection of the reinforced concrete ceiling.

The numerical verification, with or without deflections is presented in Table 7, together with comments. There are stress comparisons in selected sections to the maximum horizontal, vertical and tangential strengths of the LFS with aluminium lamellae. The stress in these sections was divided into two variants, T—thermal action without concrete ceiling deflection, T + U—thermal action and imposed load with the maximum standard concrete ceiling deflection. These two loads are separated by the “/” sign. Other sections IV–VI, included in Table 7 were chosen after analysing the stress maps—with maximum standard deflection and thermal action on the floor, but without imposed loads. This was necessary for complete stress analysis. Additionally, it was noticed that shear stresses at the edges of the floor, which was confirmed by the analytical methods for determining the stresses of glued layers described by da Silva et al. in [30,31] and Adams, Wake [32].

It is worth noting that there is no risk of chipping the cement joint between the ceramic tiles in LFS with aluminium lamellae made from BondT8 and Force polyurethane adhesives (assuming maximum deflection). It is the opposite situation to LFS without lamellae, reinforced by C2S1 adhesive with fibre mesh, described in [26], where this problem had been observed. Although, the cement joints are between the tiles, the horizontal stresses are close to the load-bearing capacity (*σ_x_* = 13.03 MPa < 15.3 MPa). It is concerning when the service loads and maximum standard deflections with thermal action value DT = 16.6 °C (40.0–23.4 °C from Table 5) between the floor and air temperature occur simultaneously.

Besides, there are large stocks of load capacity in each LFS layer with lamellae. Moreover, joining the light floor system with the concrete ceiling using C2S1 cement adhesive, the stress is lower than the load-bearing capacity of this adhesive.

Other calculation simulations, e.g., T + U with maximum deflections but without the imposed load—which are not in Table 7, confirmed that the condition of the load capacity of the floor layers and its adhesive connection with the ceiling were met (the stress was even lower than in simulations from Table 7), namely:

- The maximum compressive stress ***σ_x_*** in the tiles is ⊖ 9.42 < 240 MPa (when the imposed load was there, the maximum stress was ⊖ 16.72 MPa) and in the C2S1 adhesive (on the joints I–I) was ⊖ 4.64 < 15.3 MPa (when the imposed load was there, the maximum stress was ⊖ 13.03 MPa).
- The maximum tensile stress ***σ_x_*** in the C2S1 adhesive, which connected the concrete ceiling to the XPS thermal insulation, was ⊕ 0.03 < 15.3 MPa (when the imposed load was there, the maximum stress was ⊕ 0.10 MPa).

## 5. Conclusions

There is a lot of research in the literature on the efficiency and thermal comfort of LFS and its advantages. Taking this into consideration, the authors of this article went further and undertook to check whether the stresses of individual layers of this type of system are not greater than their strength. If so, they asked whether it can be used in a specific material application without fear of damage? This was the main reason why this type of LFS research and analysis was undertaken.

The experimental studies and computational analyses of LFS with aluminium lamellae justify the specification of the final conclusions. The applications were divided into two thematic groups according to the scope of the work, namely:

- conclusions regarding experimental research.
- conclusions regarding the numerical model.

1. Conclusions regarding experimental research:
  - The validity of locating the strain gauges, separated by polypropylene foil (to eliminate the influence of the adjacent layer on the strain gauge reading) was confirmed in the FEM calculation model, as the results were reproduced well. This confirms the correctness of the preparation and implementation of experimental research.
  - It was necessary (in the case of thermal actions) to perform correction readings (as part of subsequent research on strain gauges glued separately on each of the LFS materials), which then had to be considered in the final deformation results of each material.
  - Thanks to the use of the digital image correlation (DIG) method, the displacement result of the experimental model amounting to 0.209 mm was obtained. The correctness of the measurements was confirmed in the numerical model.
2. Conclusions regarding the numerical model:
  - Simplification of the calculation model to a plane stress state (PSS) imitates the actual boundary conditions of the floor, where expansion space is left between the floor and the walls, constituting the room boundaries, in order to allow possible thermal deformation and allows using of lower computing power while maintaining adequate accuracy.
  - Based on the comparative analysis, the stresses were not higher than the materials strength of the lightweight floor under the accepted load conditions in this research.
  - The results of numerical research show that the shear stresses of the floor between the composite layers are the highest in the edge zone.
  - Considering the results and analyses, it can be concluded that the presented lightweight floor system (LFS) with aluminium lamellae and polyurethane adhesives, with a heating coil, can be safely used.

The results of the conducted research justify its further development, such as:

(a) The LFS analysis of non-ceramic floors, e.g., glued floorboards, vinyl floors, wood floors and others,
(b) checking the strength of the system with different types and thicknesses of thermal insulation layers,
(c) analysis of the mechanical fastening of thermal insulation to the wooden substrate, which may take place in buildings with wooden structures, using various load patterns and thermal impacts.

Finally, to develop guidelines for the design and implementation of LFS in various insulation and flooring variants.

## Figures and Tables

**Table 1:** Material parameters from the above literature.

Material	Young’s Modulus *E* [MPa]	Thermal Expansion α [10^−6^/K]	Poisson’s Ratio *ν*
Ceramic tile	50,000	8	0.16
Aluminium foil	69,000	23.1	0.33
XPS 300	13–15	70–75	0.20
EPS 200	7.8	55	0.17

**Table 2:** The results of the temperature correction *Ɛ_v_* obtained based on Equation (1).

Type of the Strain Gauge Substrate	The Temperature Correction *Ɛ_v_* [μm/m] for D*T* [°C] of the Air and the Material									
	1/2	3/4	5/6	7/8	9/10	11/12	13/14	15/16	17/18	19/20
Tile	−33/ −66	−99/ −132	−165/ −198	−231/ −264	−297/ −330	−363/ −396	−429/ −462	−495/ −528	−561/ −594	−627/ −660
BondT8	40/80	120/ 160	200/ 240	280/ 320	360/ 400	440/ 480	520/ 560	600/ 640	680/ 720	760/ 800
Force	12/ 24	36/ 48	60/ 72	84/ 96	108/ 120	132/ 144	156/ 168	180/ 192	204/ 216	228/ 240
XPS	29/ 58	87/ 116	145/ 174	203/ 232	261/ 290	319/ 348	377/ 406	435/ 464	493/ 522	551/ 580

**Table 3:** The results of the temperature correction *Ɛ_v_* obtained from the experimental correction research.

Type of the Strain Gauge Substrate	The Temperature Correction *Ɛ_v_* [μm/m] for D*T* [°C] of the Air and the Material									
	1/2	3/4	5/6	7/8	9/10	11/12	13/14	15/16	17/18	19/20
Tile	−20/ −45	−85/ −110	−180/ −250	−300/ −350	−380/ −430	−475/ −500	−540/ −540	−540 −540	−540 −540	−540 −540
BondT8	−10/ −35	−65/ −100	−120/ −160	−185/ −200	−225/ −245	−270/ −285	−300/ −330	−360/ −410	−450/ −450	−450 −450
Force	−15/−30	−45/ −65	−80/ −100	−120/ −140	−160/ −180	−195/ −210	−220/ −230	−240/ −260	−285/ −310	−310 −310
XPS	−25/ −50	−85/ −100	−125/ −150	−175/ −205	−212/ −220	−235/ −250	−270/ −295	−305/ −320	−350 −350	−350 −350

**Table 4:** Results for preliminary deformations of the floor layers in polyurethane adhesive before the correction (average air temperature *T_i_* = 23.4 °C, maximum temperature of the adhesive (Force) on the sensor *T_kl_* = 40.5 °C.

Adhesives/*T_kl_/T_w_*		*T_po_*	[*T_po_ − T_i_*]	Time	Preliminary Deformations [μm/m]						
**BondT8/Force**	**[°C]**	**[°C]**	**[°C]**	**[s]**	**1*G_f_***	**2*G_k_***	**3*K_fg_***	**4*X_f_***	**5*X_k_***	**6*K_fx_***	**7*K***
	25/24.2	23.5	0.1	320	−10	**−6**	−14	−6	**−17**	−8	**−1**
	27/25	23.6	0.2	500	−23	**−24**	−23	−8	**−24**	−11	**−3**
	29/26.2	24.0	0.6	720	−43	**−61**	−32	−13	**−35**	−16	**−10**
	30/26.9	24.5	1.1	810	−51	**−64**	−35	−16	**−39**	−19	**−13**
	32/28.8	26.0	2.6	1110	−159	**−153**	−45	−28	**−52**	−27	**−25**
	33/29.2	26.5	3.1	1320	−178	**−168**	−52	−37	**−57**	−32	**−33**
	34/29.9	26.8	3.4	1600	−202	**−187**	−60	−53	**−66**	−39	**−44**
	35/30.7	27.5	4.1	2000	−232	**−212**	−70	−75	**−78**	−49	**−57**
	36/31.6	28.3	4.9	2640	−272	**−248**	−85	−111	**−98**	−64	**−78**
	37/32.6	29.2	5.8	3030	−292	**−267**	−93	−130	**−109**	−73	**−90**
	38/32.9	30.0	6.6	3810	−327	**−294**	−104	−158	**−126**	−85	**−107**
	39/33.5	30.9	7.5	4850	−357	**−317**	−112	−185	**−144**	−97	**−126**
	40/35.4	31.9	8.5	7000	−393	**−370**	−132	−213	**−163**	−113	**−147**
	40,3/35.6	32.1	8.7	8300	−401	**−380**	−132	−219	**−166**	−115	**−152**
	40,5/35.7	32.0	8.6	32,450	−406	**−413**	−98	−254	**−176**	−134	**−163**

*T_kl_*—adhesive temperature above the insulation (reading from the sensor), *T_w_*—interface temperature between the adhesive and ceramic tile, determined by the Equation (2), *T_po_*—temperature on the floor.

**Table 5:** Data of the material deformations taking the corrections of strain gauges with polyurethane adhesives (average air temperature *T_i_* = 23.4 °C, max adhesive temperature on the sensor *T_kl_* = 40.5 °C.

Adhesives/*T_kl_/T_w_*		*T_po_*	[*T_w_ − T_i_*]	[*T_kl_ − T_i_*]	Time	Final Deformations [μm/m]					
**BondT8/Force**	**[°C]**	**[°C]**	**[°C]**	**[°C]**	**[s]**	**1*G_f_***	**2*G_k_***	**3*K_fg_***	**4*X_f_***	**6*K_fx_***	**7*K***
	25/24.2	23.5	0.8	1.6	320	6	**10**	6	34	16	**16**
	27/25	23.6	1.6	3.6	500	12	**11**	10	86	46	**35**
	29/26.2	24.0	2.8	5.6	720	34	**16**	27	127	76	**65**
	30/26.9	24.5	3.5	6.6	810	47	**33**	47	149	93	**87**
	32/28.6	26.0	5.2	8.6	1110	49	**55**	91	181	125	**135**
	33/29.3	26.5	5.9	9.6	1320	58	**68**	100	180	140	**137**
	34/29.9	26.8	6.5	10.6	1600	73	**88**	112	176	150	**142**
	35/30.7	27.5	7.3	11.6	2000	83	**103**	119	170	155	**143**
	36/31.6	28.3	8.2	12.6	2640	84	**108**	120	151	152	**142**
	37/32.6	29.2	9.2	13.6	3030	98	**123**	136	158	153	**147**
	38/33.5	30.0	10.1	14.6	3810	107	**140**	131	143	151	**143**
	39/34.4	30.9	11.0	15.6	4850	118	**158**	136	129	155	**140**
	40/35.4	31.9	12.0	16.6	7000	107	**130**	153	125	162	**149**
	40,3/35.6	32.1	12.2	16.9	8300	107	**128**	156	101	168	**147**
	40,5/35.7	32.0	12.3	17.1	32,450	106	**100**	191	96	154	**137**

*T_kl_*—adhesive temperature above the insulation (reading from the sensor), *T_w_*—interface temperature between the adhesive and ceramic tile, determined by the Equation (2), *T_po_*—temperature on the floor.

**Table 6:** Comparison of deformations in LFS model—floor with lamellae and polyurethane adhesive, obtained from the numerical calculations and the experiment.

Measurement Point	Computational Deformation	Measured Deformation	Relative Difference (%)	Comments
1*G_f_*	+100.5 × 10^−6^	+107.0 × 10^−6^	6.1	Strain gauge glued to the tile, separated from the BondT8 adhesive
2*G*	+100.7 × 10^−6^	+130.0 × 10^−6^	22.5	Strain gauge on the tile, covered with adhesive
3*K_f_*	+157.7 × 10^−6^	+153.0 × 10^−6^	3.1	Strain gauge glued to the BondT8 adhesive, separated from the tile
4*X_f_*	+174.5 × 10^−6^	+125.0 × 10^−6^	39.6	Strain gauge glued to the XPS, separated from the Force adhesive
6*K_f_*	+166.5 × 10^−6^	+162.0 × 10^−6^	2.8	Strain gauge glued to the Force adhesive, separated from the XPS
7*K*	+140.7 × 10^−6^	+149.0 × 10^−6^	5.6	Strain gauge on the BondT8 adhesive, covered with this adhesive

**Table 7:** The stresses and the strength in the LFS with lamellae.

Layer	Stress in Sections (T/T + U) (MPa)		Strength(MPa)	Comments
	*σ_x_*	*σ_y_*//*τ*		
Tile [33]/ C2S1 (I)	⊖ 4.62/16.72 (V/III) ⊖ 2.55/13.03 (I)	⊖ 5.63/5.66 (VI) ⊖1.65/2.54 (I)//0.209/0.214 (VI)	⊖ 240 ⊕52.0 ⊖ 15.3	Large stocks of load capacity in ceramic tile. The *σ_x_* stress of the adhesive in the joint is close to strength, at (T + U)
BondT8	⊕ 0.04/⊖0.018 (VI/I)	⊖ 0.011/0.015 (I) //0.016/0.05 (VI)	⊖ (−); ⊕ 0.65; 0.6—*σ_ymax_* //1.0	Large stocks of load capacity in adhesive
Aluminium foil	⊖ 25.3/76.0 (I) ⊕ 3.01/ ⊖ 32.19 (VI)	-	⊖ >120⊕ 64.58	Large stocks of load capacity in the foil. No compression failure, marked by (-)
Force	⊖ 0.35/0.9 (I)	⊖ 0.54/0.56 (VI) //0.043/0.02 (VI)	⊖ (−); ⊕ 5.5; 0.65—*σ_ymax_* //9	Large stocks of load capacity in adhesive
XPS	⊖ 0.017/0.04 (III)	⊖ 0.03/0.05 (VI) //0.004/0.014 (VI)	⊖ 0.30 ⊕ 0.40//0.15	Large stocks of load capacity in XPS
C2S1	⊕ 0.03/0.10(VI/IV)	⊖ 0.007/0.02 (I/VI)//0.01/0.037 (VI)	⊖ 15.3; ⊕ 1.35; //0.4	Large stocks of load capacity in connection with the ceiling

⊕—tensile stresses, ⊖—compressive stresses, ***σ_ymax_***—max. detachment stresses, T—thermal action without deflection of the concrete ceiling, T + U—thermal action and imposed load with the maximum deflection of the concrete ceiling.

## Data Availability

The data presented in this study are available upon request from the corresponding author.

## References

[1] Suhaili S.S., Alias N.N., Mydin A.O., Awang H. (2021). Influence of oil palm spikelets fibre on mechanical properties of lightweight foamed concrete. J. Civ. Eng. Sci. Technol..

[2] Lee Y.H., Chua H., Amran M., Lee Y.Y., Kueh A.B.H., Fediuk R., Vatin N., Vasilev Y. (2021). Thermal Performance of Structural Lightweight Concrete Composites for Potential Energy Saving. Crystals.

[3] Lee Y.H., Amran M., Lee Y.Y., Kueh A.B.H., Kiew S.F., Fediuk R., Vatin N., Vasilev Y. (2021). Thermal Behavior and Energy Efficiency of Modified Concretes in the Tropical Climate: A Systemic Review. Sustainability.

[4] Liu W., Liu L., Wu H., Chen Y., Zheng X., Li N., Zhang Z. (2022). Performance analysis and offshore applications of the diffuser augmented tidal turbines. Ships Offshore Struct..

[5] Tedeschi C., Kwiecien A., Valluzzi M.R., Binda L., Zajac B. (2014). Thermal ageing and salt decay on bond between FRP and masonry. Mater. Struct..

[6] Valluzzi M.R., Garbin E., Panizza M., Binda L., Tedeschi C. Moisture and Temperature Influence on FRP Masonry Bonding. Proceedings of the XII International Conference on Durability of Building Materials and Components.

[7] Zając B., Kwiecien A. Thermal stress generated in masonries by stiff and flexible bonding materials. Proceedings of the 9th International Masonry Conference in Guimarães, ID_1629.

[8] Yang J., Fu L.-Y., Zhang Y., Han T. (2022). Temperature- and Pressure-Dependent Pore Microstructures Using Static and Dynamic Moduli and Their Correlation. Rock Mech. Rock Eng..

[9] Yang Z., Xu J., Feng Q., Liu W., He P., Fu S. (2022). Elastoplastic Analytical Solution for the Stress and Deformation of the Surrounding Rock in Cold Region Tunnels Considering the Influence of the Temperature Field. Int. J. Geomech..

[10] (2016). Ceramic Tiles Definitions, Classification, Characteristics, Evaluation of Conformity and Marking.

[11] (2014). Ceramic Tiles–Part 8: Determination of Linear Thermal Expansion.

[12] Piekarczyk W., Kata D., Lis J., Galos K. (2008). Metodyka ultra dwiękowych badań płytek typu gres porcellanato. Mater. Ceram..

[13] German J. Podstawowe Właściwości Wybranych Materiałów. Pomoce–Tablice Wybranych Kształtowników i Charakterystyk Mate-riałowych. Politechnika Krakowska. Katedra Wytrzymałości Materiałów. http://limba.wil.pk.edu.pl/~jg/.

[14] (2004). Aluminium i Stopy Aluminium–Blachy, Taśmy i płytyxwłasności Mechaniczne.

[15] (2016). Thermal Insulation Products for Buildings-Factory Made Expanded Polystyrene (EPS) Products–Specification..

[16] (2015). Thermal Insulation Products for Buildings-Factory Made Extruded Polystyrene Foam (XPS) Products-Specification.

[17] Fibran S.A., Product Catalogue, Extruded Polystyrene Thermal Insulation FIBRANxps Thessaloniki, Greece 2010. https://fibran.gr/files4users/files/documentation%20XPS/100_CATALOGUE_WEB%20[EN].pdf.

[18] (2018). Technical Data STYROFOAM™ LB-X Extruded Polystyrene Foam XPS (EN13164)—Free from HCFC—Blue Color.

[19] Elragi A.F. (2000). Selected Engineering Properties and Applications of EPS Geofoam. Ph.D. Thesis.

[20] Padade A.H., Mandal J.N. (2012). Behavior of Expanded Polystyrene (EPS) Geofoam Under Triaxial Loading Conditions. EJGE.

[21] Shulmeister V. (1997). Modelling of the Mechanical Properties of Low-Density Foams. Ph.D. Thesis.

[22] (2008). Tech Solutions 610.0 Styrofoam™ Brand Extruded Polystyrene Insulation for Lightweight Fill Application.

[23] Karpiesiuk J. (2020). Mechanical Strength Indicators of Polyurethane Adhesive in Lightweight Floor Systems. Tech. Trans..

[24] Karpiesiuk J. (2020). Young’s modulus and Poisson’s ratio of Polyurethane Adhesive in Lightweight Floor System. Mod. Approaches Mater. Sci..

[25] Karpiesiuk J., Chyzy T. (2021). Analysis of Deformation and Stresses of a Lightweight Floor System (LFS) under Thermal Action. Materials.

[26] Hoffmann K. (1989). An Introduction to Measurements Using Strain Gauges.

[27] Gayevoy A.V., Lissel S.L. Strain reading correction for apparent strain and thermal expansion coefficient of masonry and brick. Proceedings of the 10th Canadian Masonry Symposium.

[28] Zajac B. (2018). Ścinane Połączenia Klejone Sztywne i Podatne Pracujące w Podwyższonej Temperaturze.

[29] (2008). Eurocode 2: Design of Concrete Structures—Part 1-1: General Rules and Rules for Buildings.

[30] Da Silva L.F.M., das Neves P.J.C., Adams R.D., Spelt J.K. (2009). Analytical models of adhesively bonded joints—Part I. IJAA.

[31] Da Silva L.F.M., das Neves P.J.C., Adams R.D., Wang A., Spelt J.K. (2009). Analytical models of adhesively bonded joints—Part II. IJAA.

[32] Adams R.D., Wake W.C. (1984). Structural Adhesive Joints in Engineering.

[33] (2016). Badania Laboratoryjne Wyrobu Gotowego–Gres TERO, gr. 8,5 mm.

